# A beaker method for determination of microplastic concentration by micro-Raman spectroscopy

**DOI:** 10.1016/j.mex.2023.102251

**Published:** 2023-06-15

**Authors:** Zijiang Yang, Hisayuki Arakawa

**Affiliations:** Department of Ocean Sciences, Tokyo University of Marine Science and Technology, Konan 4-5-7, Minato-Ku, Tokyo 108-8477, Japan

**Keywords:** Raman spectroscopy, Microplastics, Small microplastic marine pollution, A beaker method for determination of microplastic concentration by micro-Raman spectroscopy

## Abstract

Fourier-transform infrared (FT-IR) spectroscopy method for measuring small microplastic (SMP) concentration in marine environment is time-consuming and labor-intensive due to sample pre-treatment. In contrast, Raman spectroscopy is less influenced by water and can directly measure SMP samples in water, making it a more efficient method to measure SMP concentration. Therefore, a method that can directly estimate the concentration of SMPs in water was developed, and the relationship between SMP concentration and experimental Raman spectra were established by testing with standard polyethylene (PE) samples. It was found that average spectra acquired in water solution could reflect characteristic peaks of the plastic after baseline correction. Further investigation found that there is a significant functional relationship between correlation coefficient of sample spectra and the concentration of PE particles, and such relationship can be modelled by Langmuir model. The empirical functional relationships can be used to estimate SMP concentrations by measuring average Raman spectra. The developed methodology is helpful for developing rapid SMP identification and monitoring methods in a more complex manner.

• A method of directly measuring MP concentration in water is proposed.
• Experimental procedures are provided.
• Data analysis methods are outlined.

## Graphical abstract

**Figure:**
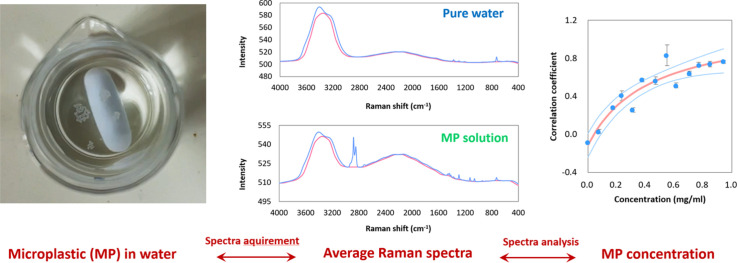

Specifications table

**Table:** 

Subject area:	Environmental science
More specific subject area:	*Detection and measurement of microplastics*
Name of your method:	*A beaker method for determination of microplastic concentration by micro-Raman spectroscopy*
Name and reference of original method:	*N.A.*
Resource availability:	*Excel spreadsheet and Matlab script:*https://github.com/River20104047/Beaker_Method

## Method details

### Background

Small microplastics (SMPs), categorized as particles with diameters less than 1 mm, pose an escalating threat to marine environment [1], [2], [3], [4], [5]. SMPs can pose higher risk in comparison to their larger counterparts, large microplastics (LMPs, defined as particles with diameters between 1 mm and 5 mm), due to their higher likelihood of ingestion by a wide array of marine organisms, attributed to their diminutive size [6]. For example, SMPs are found in various marine species [7], [8], [9], [10], [11], [12]. The assimilation of SMPs by these organisms can incite numerous negative effects, such as physical damage [13] and inflammation [14]. Some studies also report the negative impact on reproductive success, specifically, diminished hatching rates [15] and survival rates [16] of fish. Furthermore, SMPs can act as carriers for harmful chemicals [5,17], pathogens, and invasive species [18]. The potential for SMPs to permeate the food chain may ultimately result in negative effects on human health [19]. Therefore, the detection and monitoring of SMPs are important to better understand the behavior, environmental fate, and dynamic process of SMPs.

Raman micro-imaging and Fourier transform infrared (FTIR) micro-imaging constitute the conventional methodologies for quantifying SMPs. These techniques provide non-destructive analyses, preserving the integrity of the samples [20,21]. The micro-imaging procedure typically involves the identification of potential SMPs during an initial scan, followed by a more in-depth spectral analysis of these identified particles during a subsequent pass [22], [23], [24], [25], [26], [27]. Nevertheless, this method requires a large number of spectra over small areas to achieve high-resolution images, which results in a lengthy measurement period, rendering the process labor intense and time-consuming [28,29].

When conducting certain assessments of SMPs in the ocean, a generalized approximation of the plastic type and concentration might be all required, with the size of SMPs being less critical. This cursory estimate can provide valuable information that aids in the formulation of more nuanced and detailed experimental designs and sample processing plans. Given the relative insensitivity of Raman spectroscopy to water [30], this allows SMP samples to be directly measured in the aqueous phase. Thus, it would be highly beneficial to devise a method that capitalizes on these specific attributes of Raman spectroscopy, thereby facilitating rapid and efficient measurements of SMPs in marine environments.

In this study, we present a new experimental approach that enables direct measurement of SMP samples in water. Subsequently, we also put this proposed approach to the test by gaging its feasibility for estimating SMP concentrations using standard SMP samples. We posit that this methodology could serve as a precursor for future in-depth investigations into the spectral analysis inherent to this approach.

### Preparation

#### Instrument

**Fig. 1:**
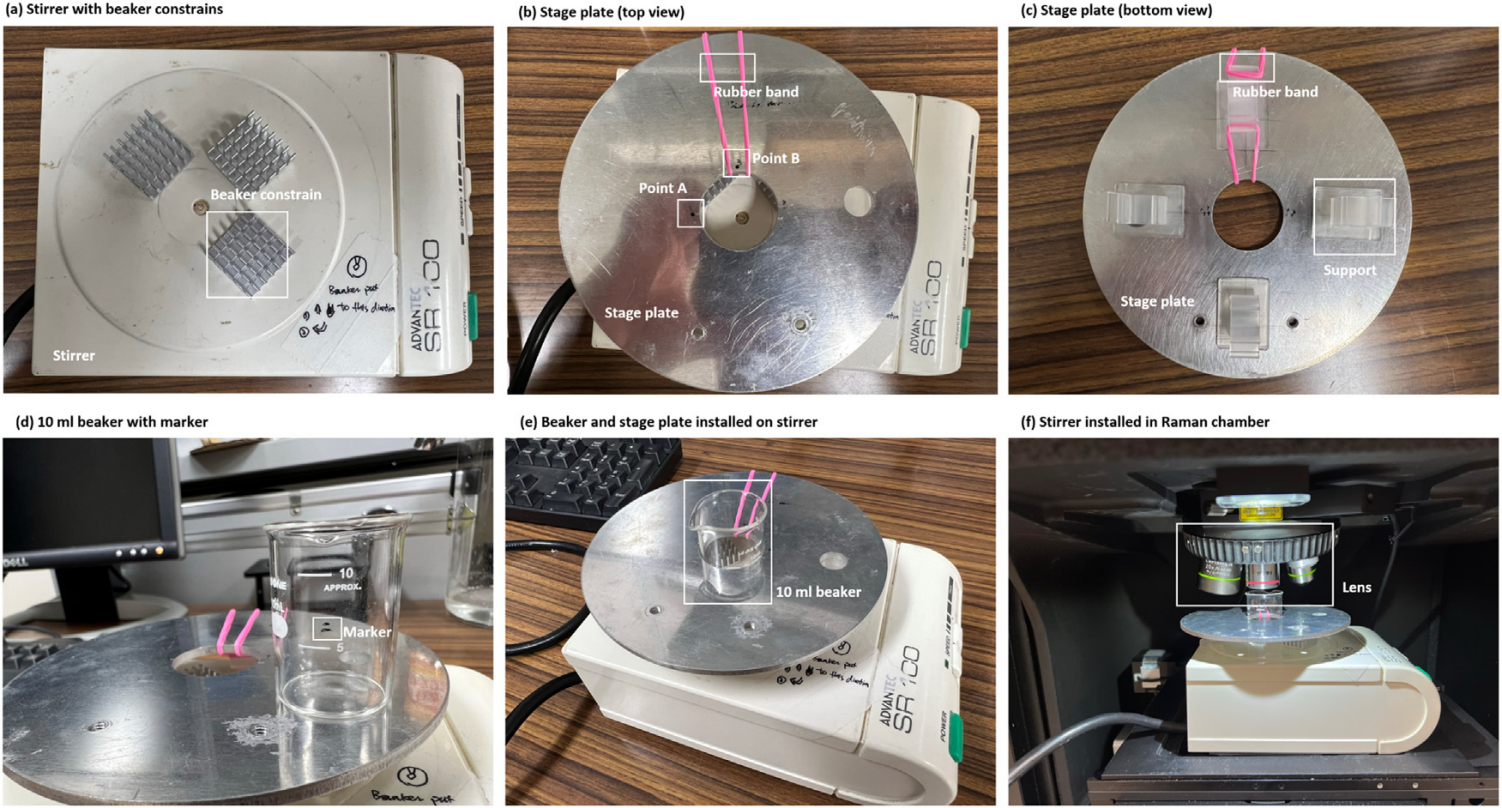
Photos of instruments.

Aside from the Raman spectrometer, additional laboratory instruments are required to execute the experiment. A comprehensive overview of the tools utilized in this study is depicted in Fig. 1. To conduct the measurements, the subsequent instruments are essential.

1. Stirrer that can fit in Raman spectrometer chamber (Fig. 1a). In this study, a small stirrer (SR-100, SANSYO Inc, Japan) was used.
2. Some constraints and supports to fix the position of the beaker (Fig. 1b and c). In this study, 20 × 20 × 6 mm (*L* × *W* × *H)* aluminum heat sink units were used for constraints, and 25 × 25 × 18 mm cable holders were used as supports.
3. Small beaker that can fit within the chamber (Fig. 1d–f). In this study, 10 ml beaker (AS ONE Corporation, Japan) with size of 25 × 22.5 × 36 mm (outer *D* × inner *D* × *H)* was used.

#### Markers for locating

**Fig. 2:**
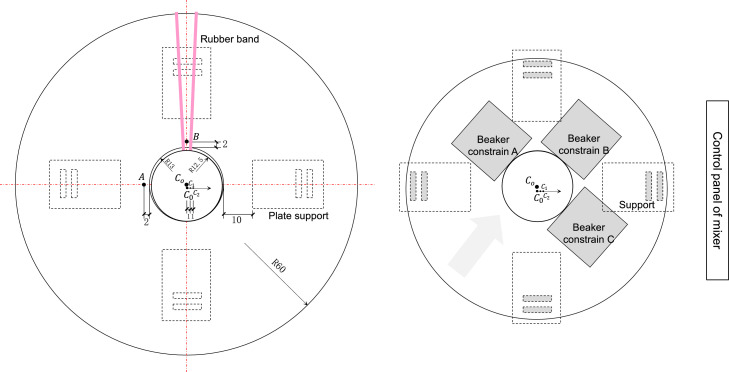
Geometrical configuration of aluminum plate, beaker, and supports. Unit in the figure is [mm].

Geometrical markers are incorporated onto the stage plate to designate the center of the beaker, identified as Point A and Point B (Figs. 1b and 2). These reference points serve to pinpoint the beaker's center. In our study, we employed a microscopic stage plate, secured with a rubber band, to facilitate this centering process. Given the measurable parameters such as the stage and beaker dimensions, along with the relative coordinates of Points A and B, the beaker's center can be geometrically determined.

Given the confined space within the chamber, the optimal water surface height within the beaker is ascertained by adjusting the focal plane of the lens. Once this optimal height is determined, markers are added to the beaker. In this study, two boundary lines were marked, denoting the upper and lower limits of the water surface caused by capillary attraction. In this context, the marker corresponds to 6.37 ml of water, as measured by the water mass (Fig. 1d), and the distance between the lens and water surface approximately measures 25 mm.

#### Rotational speeds of stirrer

Two distinct rotational speeds of the stirrer are established for this experiment. Speed I refers to the slowest velocity capable of driving the rotation of the stir bar, whereas Speed II signifies a faster velocity that can agitate the water without causing any spillage. During the Raman spectra measurement, Speed I is utilized to minimize any distortion of the water surface. Based on visual observation, the perturbation of the water surface under Speed I is minimal. Conversely, Speed II is employed to validate whether the stir bar rotates around the beaker's center. An illustrative video, which demonstrates the water surface under both Speed I and Speed II, can be accessed online (https://youtu.be/pTPQ-Sp1a1U).

### Procedures

Part 1 *Weighting samples*

1. Measure a specific quantity of SMP particles on a scale with 0.1 mg accuracy using weighing paper.
2. Carefully transfer the SMP particles to the beaker, rinsing with distilled water.
3. Place the stir bar into the beaker and add more DI water until the water surface reaches the established marker (6.37 ml). This volume has been selected to optimize the height given the limited space within the Raman micro-spectrometer chamber.
4. Insert the beaker onto the stage plate and cover it with a Petri dish to prevent contamination.

Note: Be mindful of the volume of water used when rinsing with weighing paper. Ensure that the rubber band does not obstruct Point B (See Fig. 2).

Part 2 *Installing the beaker*

5. Launch the Raman software and device. Connect the stirrer to a power source.
6. Position the stirrer on the stage inside the Raman spectrometer (Fig. 1f). Ensure the control panel of the stirrer is oriented towards the right side. Adjust its positioning to align its center directly underneath the lens sight area.
7. Place the aluminum plate (containing the beaker) onto the stirrer and adjust the plate's position with the beaker. During this process, adhere to the following considerations:
  (1) Ensure the rubber band, Point A, and Point B are in the relative positions depicted in Fig. 2. The side with the rubber band should face the interior of the chamber.
  (2) Adjust the beaker position to ensure it is adjacent to beaker constraints A and B.
  (3) Gently rotate the plate counterclockwise until its position is constrained by the beaker constraints.
8. Confirm the use of a 5 × lens.
9. Turn on the stirrer, adjust the mixing speed to Speed II (faster mode) and make sure there is no noise from collision of stir bar and beaker wall.

Note: Make sure no SMP particles assemble into big chunks and sink to the bottom.

Part 3 *Locating measurement points*

10. Move the microscopic stage until Point A is under the lens. Then, under microscopic view, manually identify the center of Point A and record its coordinates: *X*_A_, *Y*_A_.

Note: Exercise caution during this process to ensure that the 5 × lens does not contact the beaker.

**Fig. 3:**
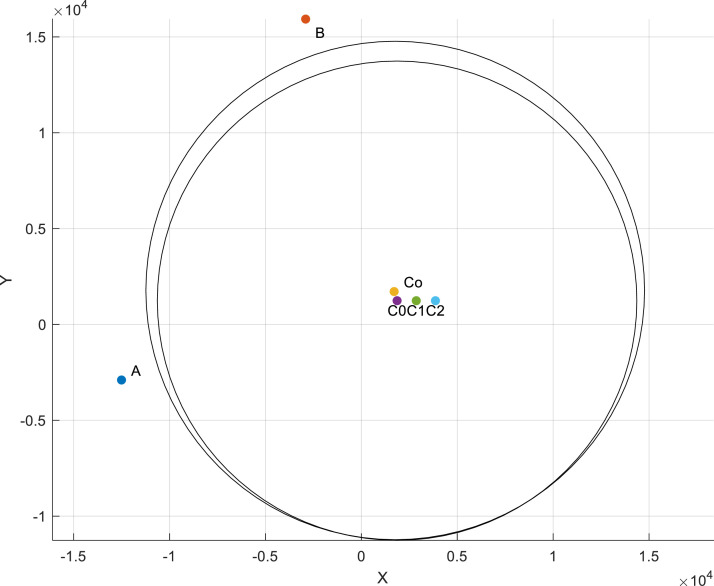
Matlab generated figure for checking positions. In the figure, the large circle refers to top view of inner hole of the plate (*r* = 13 mm) and the small circle refers to top view of beaker (Fig. 2). A and B represent Point A and Point B, Co is the center of plate, C_0_ is the center of the beaker, and C_0_, C_1_, C_2_ are the three sampling points. Unit of *x*-axis and *y*-axis is µm.

11. Tracing along the edge of the beaker, move upward and to the right until Point B is encountered. Then, manually locate the center of Point B and record its coordinates, *X*_B_, *Y*_B_.
12. Utilize the coordinate calculator Matlab script (https://github.com/River20104047/Beaker_Method) to estimate the positions of the plate center (C_o_) and beaker center (C_0_) using the recorded coordinates [*X*_A_, *Y*_A_; *X*_B_, *Y*_B_]. Execute the code to obtain [${\hat{X}}_{o},\;{\hat{Y}}_{o};\;{\hat{X}}_{0},\;{\hat{Y}}_{0}$]. The computation is based on the geometric relationships between the plate, beaker, Point A, and Point B (Fig. 2).
13. Refer to the Matlab figure to check the relative position accuracy (Fig. 3). C_o_ should be positioned between Point B and C_0_.

**Fig. 4:**
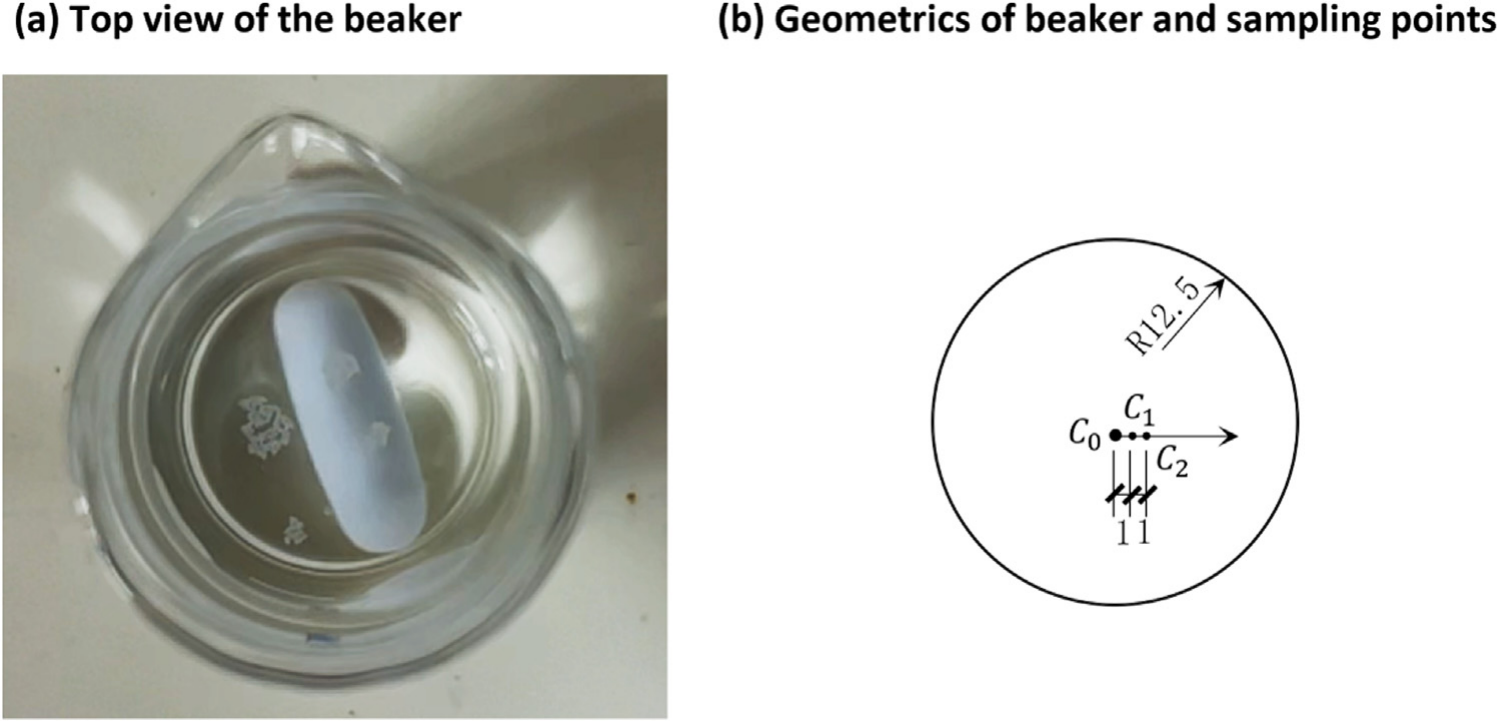
Demonstration of the sample and measuring points. The picture in (a) showed when 3.0 mg PE particles were added to the beaker, i.e., 0.47 mg/ml. *C*_0_, *C*_1_ and *C*_2_ represent the 3 measuring points, and the unit is in (mm).
14. Move the lens to the estimated position of C_0_, adjust the stirrer speed to Speed I (the slower mode), close the Raman chamber door, and if necessary, fine-tune the lens position to ensure the center aligns with the whirlpool center. Subsequently, record its coordinates, *X*_0_, *Y*_0_.
15. Open the chamber, switch off the stirrer, close the chamber, and adjust the lens height (*z*-axis value) to focus on the water surface. Then, record its coordinates, *Z*_0_.
16. Open the chamber, power on the stirrer, and adjust to Speed I.

Part 4 *Raman setup and measurement*

17. Ensure the use of a 5 × lens, an exposure time of 0.1 s, and 2 accumulations. Subsequently, select interval analysis, set a 10 × 8000 µm slit, schedule 1000 spectra for each measurement, and stipulate a 0.001 s interval between measurements.
18. Following the coordinate calculator Excel file (https://github.com/River20104047/Beaker_Method), input 3 sampling points as follows: C_0_ = (*X*_0_, *Y*_0_, *Z*_0_), C_1_ = (*X*_1_, *Y*_1_, *Z*_1_), C_2_ = (*X*_2_, *Y*_2_, *Z*_2_). These coordinates are derived based on the geometric relationships depicted in Fig. 2.
19. Execute three measurements at each sampling point, and then initiate the measurement. In total, nine measurements are conducted, taking approximately 45 min in total.

Note: Upon completion of all measurements, check for the formation of particle clumps. If present, make a note of it.

20. Save the data directly in the software, and subsequently export the nine measurement files into a Comma-Separated Values (CSV) format for further analysis.

Part 5 *Tentative data analysis framework*

21. For each measurement, the average of the 1000 spectra is calculated as follows:
  (1) $${\bar{y}}_{Cj-m}=\sum_{i=1}^{1000}y_{Cj-m,i}$$

where $y_{Cj-m,i}$ represents a single Raman spectrum of the 1000 spectra during the measurement, and *i* = 1,2,3…1000, *Cj* = *C*_0_, *C*_1_, *C*_2_, and *m* = 1, 2, 3 refers to the index of measurement at each sampling point. ${\bar{y}}_{Cj-m}$ represents the average spectrum of the *m*-th measurement at *Cj*. As a result, 9 average spectra are obtained for each PE concentration. Then, the average spectra are smoothed by moving average with 11 data unit [31].

22. *R*_b_, correlation between average baseline corrected spectrum and standard PE spectrum, is calculated by:
  (2) $$R_{b|Cj-m}=\frac{\sum \left(x_{s}-\bar{x}\right)\left({\bar{y}}_{b|Cj-m,s}-{\bar{\bar{y}}}_{b|Cj-m}\right)}{\sqrt{\sum {\left(x_{s}-\bar{x}\right)}^{2}\sum {\left({\bar{y}}_{b|Cj-m,s}-{\bar{\bar{y}}}_{b|Cj-m,s}\right)}^{2}}}$$

where $R_{b|Cj-m}$ is the correlation coefficient between baseline corrected spectrum and the standard PE spectrum of the *m*-th measurement (*m* = 1,2,3) at sampling point *Cj* (*Cj* = *C*_0_, *C*_1_, *C*_2_). ${\bar{y}}_{b|Cj-m,s}$ is the baseline corrected ${\bar{y}}_{Cj-m,s}$, which is calculated by:

(3) $${\bar{y}}_{b|Cj-m,s}={\bar{y}}_{Cj-m,s}-{\hat{y}}_{b,s}$$

where ${\hat{y}}_{b,s}$ is the estimated baseline at Raman shift *s*. ${\bar{\bar{y}}}_{b|Cj-m}$ is the mean of ${\bar{y}}_{b|Cj-m,s}$ along *s*. The baseline correction is conducted by applying the moving window method [32].

23. A modified Langmuir model is used to fit the relation between *R*_b_ and *C*_PE_:
  (4) $$R_{b}=\frac{b_{1}b_{2}C_{\text{PE}}}{1+b_{2}C_{\text{PE}}}+c$$

where *b*_1_, *b*_2_, *b*_3_ are parameters of the original Langmuir model, and *c* is the modification parameter for potential negative *R*_b_ values. Then, the relationship between average spectrum and concentration is established.

### Method validation

#### Sample preparation

Standard SMP samples consisting of polyethylene (PE) particles with diameters ranging from 90 to 106 µm (Cospheric Inc., USA) were dispersed in distilled water at varying concentrations. In this study, PE particle concentrations of 0 mg, 0.5 mg, 1.0 mg, 1.5 mg, 2.0 mg, 2.5 mg, 3.0 mg, 3.5 mg, 4.0 mg, 4.5 mg, 5.0 mg, 5.5 mg, and 6.0 mg were introduced into 6.37 ml of distilled water in a 10 ml beaker (AS ONE Corporation, Japan). These produced concentrations ranging from 0 to 0.94 mg/ml. The 6.37 ml volume was chosen as the height of the corresponding water surface best fit the limited space available in the chamber of the micro-Raman spectrophotometer (NRS-4500, JASCO Inc., Japan). Subsequently, the PE suspension in the beaker was transferred into the Raman micro-spectrophotometer chamber.

#### Experimental setup

A magnetic stirrer (SR-100, SANSYO Inc, Japan) was chosen due to its compact size that could be accommodated within the chamber. The PE suspension was stirred using a small magnetic stir bar at the lowest possible speed to ensure a uniform water surface. As the stirrer did not indicate the rotational speed on its panel, this was determined by frame-by-frame analysis of a recorded video (150 fps). The recording was obtained with a camera positioned directly above the beaker, with its center aligned with the beaker's center to minimize image distortion caused by lens curvature. Particle motion was also examined based on the recorded video featuring 0.47 mg/ml PE particles. During the measurements, the center of the beaker was adjusted to coincide with the rotational center of the stir bar.

#### Raman micro-spectroscopic analysis

Considering the constant upward movement and assembly of particles at the water surface even during stirring, Raman spectra were acquired as point measurements on the water surface. Moreover, given the challenge in accurately locating the center of the beaker and the variations in the whirlpool of water flow within the beaker, we chose to use three sampling points around the center to gain a more robust measurement. These three sampling points were (1) the center of the beaker (C_0_), (2) 1 mm away from the center (C_1_), and (3) 2 mm away from the center (C_2_) (Fig. 2). Due to the limited space within the chamber, a 5 × objective was employed.

At each sampling point, three sets of 1000 Raman spectra were obtained within a wavenumber range of 400 to 4000 cm^−1^ with 0.1 s exposure, using a 532 nm excitation laser, 41.4 W laser power, a 10 × 8000 µm slit, and 2 accumulations. This setup was chosen as it offered a balance between measurement time and spectral quality. A standard PE spectrum was also obtained by measuring a standard PE sample (Scientific Polymer Products Inc., USA) using the same Raman configuration but with a 2 s exposure.

#### Modeling relation between potential spectra-related variables and concentration

Preliminary assessment results suggested that *R*_b_ served as the best variable for concentration estimation. Consequently, we further explored the relationship between *R*_b_ and PE concentrations (denoted as *C*_PE_). Given the behavior of PE particles in water was akin to the surface absorption process, surface absorption isotherm models were employed to fit the dataset [33]. The isotherm models are presented in Table 1. Aside from the linear model, all other models were modified to account for the potential negative value of *R*_b_. Hence, a parameter *c* was added, which represents the *R*_b_ value when the PE concentration is zero. The goodness of fit was evaluated using mean squared error (*MSE*), root mean squared error (*RMSE*), Akaike information criterion (*AIC*), Bayesian information criterion (*BIC*), and adjusted *R*^2^ [34,35]. For reference purposes, *R*^2^ was also calculated.

**Table 1:** Summary of the surface absorption models. *C*_PE_ represents PE concentration in the beaker (mg/ml), and *R*_b_ represents correlation coefficient between baseline-corrected average spectrum (*n* = 1000) and standard PE spectrum. *b*_1_, *b*_2_, *b*_3_ represents original parameters of the model, and *c* is the modification for potential negative *R*_b_ values.

	Form	*MSE*	*RMSE*	*R*^2^	*R*_adj_^2^	*AIC*	*BIC*
Linear model	$R_{b}=b_{1}C_{\text{PE}}+b_{2}$	0.019	0.139	0.787	0.768	−12.6	−11.5
Freundlich model	$R_{b}=b_{1}{C_{\text{PE}}}^{1/b_{2}}+c$	0.013	0.115	0.867	0.841	−16.8	−15.1
Redlich-Peterson model	$R_{b}=\frac{b_{1}C_{\text{PE}}}{1+b_{2}{C_{\text{PE}}}^{b_{3}}}+c$	0.012	0.111	0.888	0.851	−17.0	−14.8
Sips model	$R_{b}=\frac{b_{1}{C_{\text{PE}}}^{b_{3}}}{1+b_{2}{C_{\text{PE}}}^{b_{3}}}+c$	0.012	0.111	0.889	0.852	−17.1	−14.8
Modified Temkin model	$R_{b}=b_{1}\ln\left(b_{2}C_{\text{PE}}+b_{3}\right)+c$	0.012	0.109	0.881	0.858	−18.2	−16.5
Dubinin-Radushkevich model	$R_{b}=b_{1}e^{-b_{2}\ln\left(1+1/C_{\text{PE}}\;\right)}+c$	0.012	0.109	0.880	0.856	−18.1	−16.4
Langmuir model	$R_{b}=\frac{b_{1}b_{2}C_{\text{PE}}}{1+b_{2}C_{\text{PE}}}+c$	0.011	0.107	0.886	0.863	−18.7	−17.0

#### Sensitivity of the method

Each measurement in our experimental setup takes about 5 min, leading to a total of 45 min for the nine measurements. In practice, this duration could be too lengthy for continuous measurements of a 6.7 ml sample. Therefore, we also conducted an analysis based solely on the spectrum obtained at C_0_ to investigate the sensitivity of the developed method with respect to the number of measurements. Subsequently, we compared *R*_b_ from the spectra obtained from the center of the beaker (noted as *R*_b|_*_C_*_0_) with *R*_b_ from the spectra obtained from the three sampling points *C*_0_, *C*_1_, and *C*_2_.

#### Baseline correction and average spectrum

**Fig. 5:**
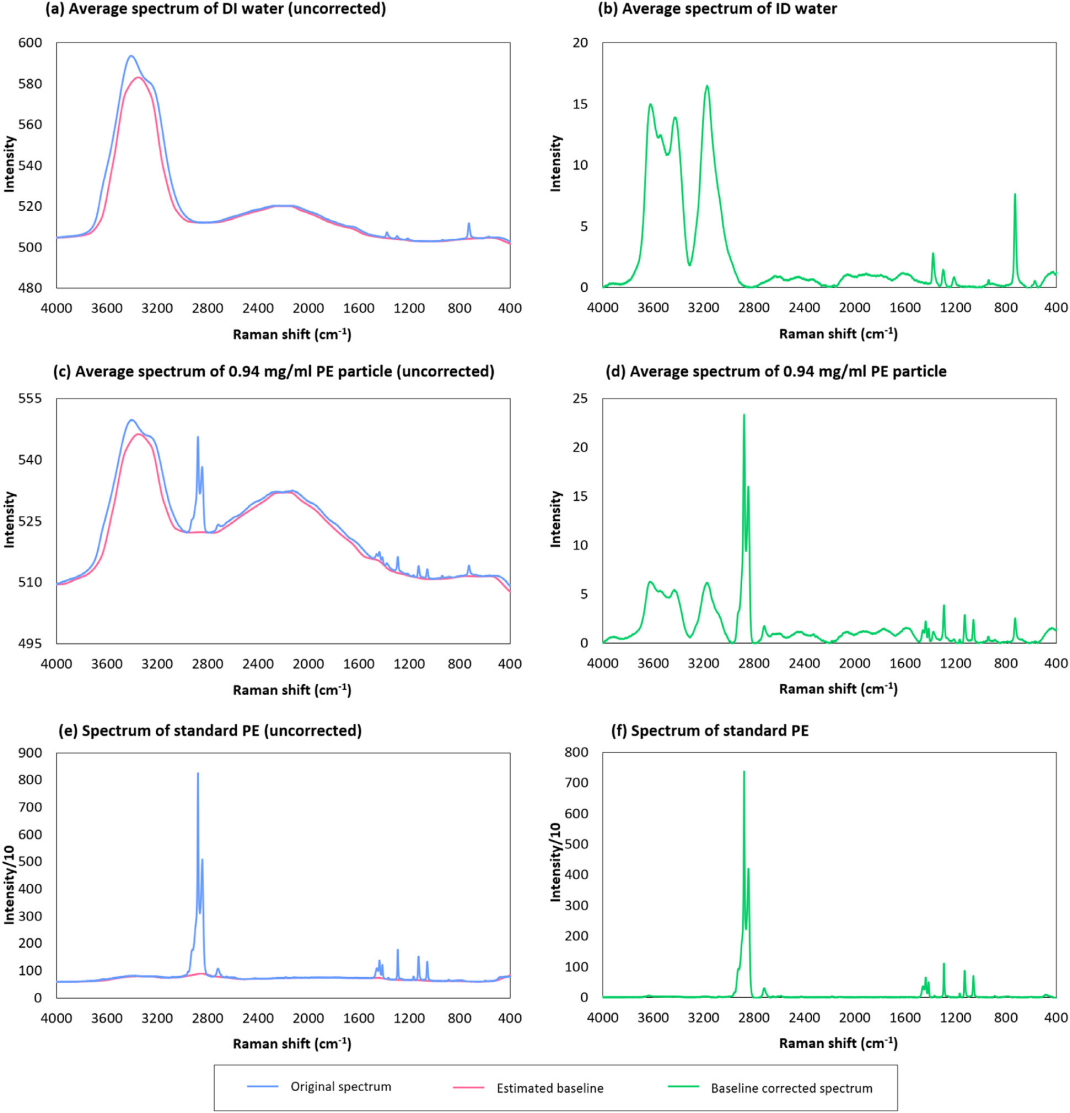
Selected Raman spectra for demonstration. (a) and (b): distilled water, i.e., 0.0 mg/ml PE. (c) and (d): 0.94 mg/ml PE. (e) and (f): standard PE in air. The data of (a), (b), (c), (d) are from sampling point *C*_1_ (Fig. 2).

An average spectrum form of distill water, i.e., 0.0 mg/ml PE, is shown in Fig. 5a and b, and an average spectrum of 0.94 mg/ml PE particles is shown in Fig. 5c and d. The results reveal that the estimated baseline generally captures the shape of the background, and the characteristic peaks of PE become more prominent after baseline correction. For the standard PE spectrum, which originally does not have interference from other materials, the baseline correction shows negligible effects on its shape (Fig. 5e and f).

The spectra reveal a distinct transformation in their shape as the concentration of PE increases, particularly the peak in the range of 2800 - 3000 cm^−1^. Consequently, the average spectrum with higher PE concentrations begins to resemble the standard PE spectrum (Fig. 5a, c, e), leading to a higher correlation coefficient. Such a difference becomes more apparent after baseline correction (Fig. 5b, d, f). Interestingly, the baseline itself also appears to be influenced by PE concentration. As PE concentration increases, the height difference between the hump in the range of 3000 ∼ 2800 cm^−1^ decreases (Fig. 5a and c).

Overall, our findings indicate that changes in PE concentration in the water can be reflected by the average spectrum transformation in shape.

#### Relation between R_b_ and concentration

Given that the assembly of PE particles at the water surface resembles a surface absorption process, we used absorption isotherm models to further investigate the relation between concentration and *R*_b_. The summary statistics of goodness-of-fit are presented in Table 1. Since models have different numbers of parameters, *R*^2^ is not a suitable parameter for comparison, and thus was not used for evaluation. Among all models, the Langmuir model shows the best performance, indicated by smaller *MSE, RMSE, AIC*, and *BIC*, and larger *R*_adj_^2^ values. Conversely, the performance of the linear model is unsatisfactory. As discussed earlier, the addition of more PE particles to the suspension will lead to surface saturation, causing the average spectrum to reach a limiting shape. Thus, *R*_b_ is expected to approach a maximum value with increasing concentration, a phenomenon that cannot be accurately represented by a linear model.

**Fig. 6:**
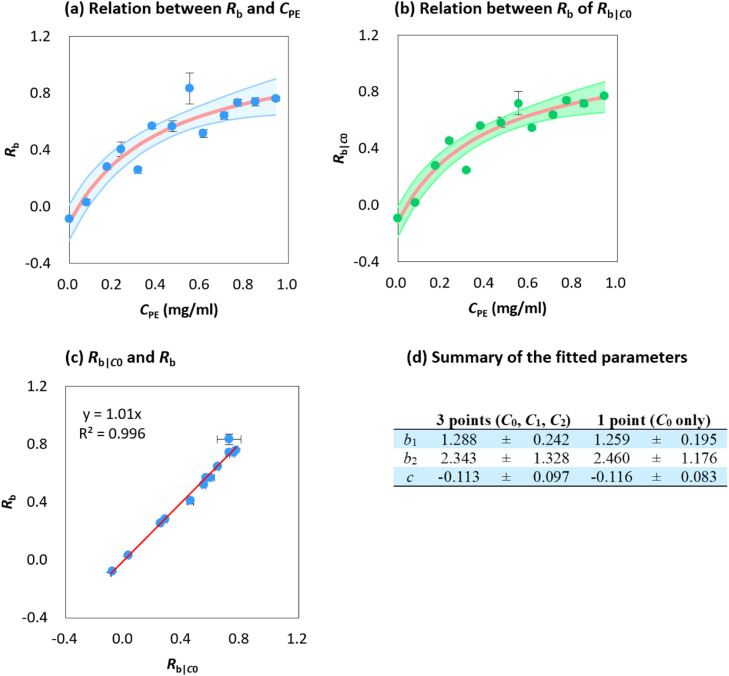
Plot of *R*_b_ and *R*_b|_*_C_*_0_ versus concentration of PE (*C*_PE_) with 95% prediction interval [36], and *R*_b_ versus *R*_b|_*_C_*_0_. Error bar in (a), (b) and (c) shows the standard deviation (*n* = 9 for *R*_b_, *n* = 3 for *R*_b|_*_C_*_0_). For (d), estimated parameters were expressed in mean ± se.

The Langmuir model, which presents the best performance, is used to model the relationship between *R*_b_ and *C*_PE_ (Fig. 6a). The estimated parameters of the model are presented in Fig. 6d. According to the Langmuir model (Table 1), *b*_1_ represents the limiting correlation coefficient when concentration approaches infinity. Theoretically, the correlation coefficient cannot be greater than one. However, we accept this result as valid since, when *C*_PE_ becomes very large, the PE particles start to form chunks and violate the assumption of the surface absorption process. Thus, these results can be considered as the best representation of the tested concentration range.

#### Sensitivity of the model to sampling points

We investigated the linear relationship between *R*_b_ based solely on spectra obtained at *C*_0_ and *R*_b_ based on all three sampling points C_0_, C_1_, C_2_, with the result presented in Fig. 6b. This shows a similar data pattern for both three measurements and nine measurements. Additionally, the estimated model parameters from three measurements fall within one standard error of the nine measurements (Fig. 6d). Further investigation found a strong linear correlation between *R*_b_ and *R*_b|_*_C_*_0_ (Fig. 6c). Thus, we conclude that the model and method are not highly sensitive to the sampling points. If time is a constraint, measurements could be performed solely at C_0_. This could increase the efficiency of the method without significantly impacting the accuracy of the results.

### Additional information

#### Movement of particles in beaker

During sample preparation, it was observed that once PE particles were mixed with water, they moved upwards to the surface, with moderate stirring unable to draw particles into the water. Strong mixing could create a uniform suspended solution, but this also damaged the particles, crushing them into smaller pieces. The floating of PE particles can be explained by their lower density (*ρ* = 0.96 g/cm^3^) compared to water. Moreover, due to the hydrophobicity of the PE particle surface, an air film may form on the surface of the particles, enhancing their floating ability. Currently, there are no efficient methods known to remove air films from the particle surface, rendering the creation of uniform PE suspensions in water impractical. Therefore, the experiment was conducted under the assumption that all SMP particles assembled at the water surface.

As the particles floated on the surface, we observed that they did not spread uniformly on the water surface, but rather assembled into clusters with the thickness of a single layer of PE particles (Fig. 4a). As the concentration increased, the size of the clusters also increased, eventually covering the entire surface area of the beaker. In addition, with an increase in concentration, a large chunk of particles with multilayer thickness might form and stay at the bottom edge of the beaker, a result of hydrophobicity of the plastics.

**Fig. 7:**
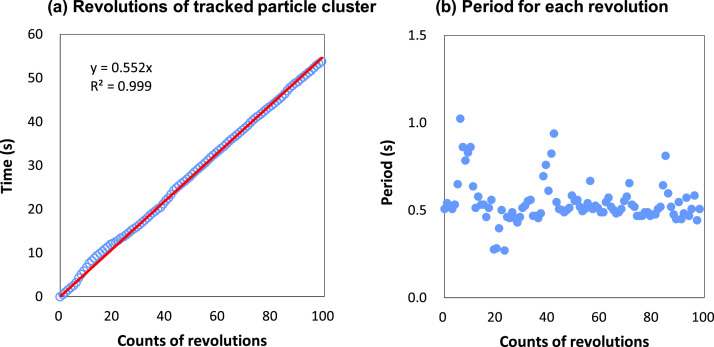
Relation between spinning and time.

The movement of these clusters was further analyzed based on a recorded video (https://youtu.be/pmTv3V3FwUE). It was observed that the clusters moved with the mixed water driven by the stir bar. However, the clusters did not follow a single orbit, but could alternate between orbits of different sizes. This orbit fluctuation resulted in fluctuations of the clusters angular speed. The relationship between revolution counts and time of the tracked cluster is shown in Fig. 7. Here, a longer period corresponds to the orbit closer to the interior wall of the beaker, while a shorter period corresponds to the orbit closer to the center of the beaker. Based on 100 continuous revolution counts from the video, we estimated the stir bar's angular speed at 13.6 rad/s (130 rpm), while the average angular speed of a tracked cluster was 11.4 rad/s (109 rpm).

#### Correlation between potential spectra-related variables and concentration

In this study, various variables were calculated from the average spectrum to estimate polyethylene (PE) concentration. The variables considered include.

*R*, correlation coefficient between the average uncorrected spectrum and the standard PE spectrum. It is calculated using the following equation [37]:

(5) $$R_{Cj-m}=\frac{\sum \left(x_{s}-\bar{x}\right)\left({\bar{y}}_{Cj-m,s}-{\bar{\bar{y}}}_{Cj-m}\right)}{\sqrt{\sum {\left(x_{s}-\bar{x}\right)}^{2}\sum {\left({\bar{y}}_{Cj-m,s}-{\bar{\bar{y}}}_{Cj-m,s}\right)}^{2}}}$$

where $R_{Cj-m,s}$ is the correlation coefficient between uncorrected spectrum and the standard PE spectrum of the *m*-th measurement (*m* = 1,2,3) at sampling point *Cj* (*Cj* = *C*_0_, *C*_1_, *C*_2_). *x_s_* is the Raman shift of the spectrum, *s* = 400, 401…4000, $\bar{x}$ is the mean of *x_s_*. ${\bar{y}}_{Cj-m,s}$ is the ${\bar{y}}_{Cj-m}$ at *s*, and ${\bar{\bar{y}}}_{Cj-m}$ is the mean of ${\bar{y}}_{Cj-m,s}$ along *s*.

*R*_b_, correlation between average baseline corrected spectrum and standard PE spectrum, which is calculated by Eqn. (2).

Other potential variables, *H*_b_ @ *k*, peak height of at different Raman shift, (*k* = 1059, 1128, 1239, 1415, 1438, 2844, 2878 cm^−1^), *f*_b_, frequency of detecting a PE particle, which is defined as the ratio of spectra with *R*_b_ > 0.2 within 1000 measurements, and *m*_b_, average *R*_b_ of spectra with *R*_b_ > 0.2, are also calculated based on baseline-corrected spectrum. Subscript *b* means that these variables are calculated from baseline-corrected spectrum.

The inter-correlation between PE concentrations and these potential variables is shown in Table 2. The results indicate that PE concentration is significantly and positively correlated with all potential variables (*p* < 0.05), which aligns with expectations. As the PE concentration increases, both the frequency of detecting PE and the average spectrum similarity to the standard PE spectrum increase. Furthermore, all potential variables exhibit significant inter-correlation (*p* < 0.05). However, due to potential variable collinearity, *R*_b_ is chosen for modeling the concentration relationship, as it best reflects overall spectrum changes. The peak heights (*H*_b_ @ *k*) may be useful for suspensions containing multiple types of microplastics.

**Table 2:** Inter-correlation coefficient between concentration and potential variables. *C*_PE_ represents concentration of microplastics. *R* is the correlation coefficient between uncorrected average spectrum and standard PE spectrum, and *R*_b_ is the correlation coefficient between baseline corrected average spectrum and standard PE spectrum. *H*_b_ represents peak height at different Raman shifts. *f*_b_ is the ratio of spectra with *R*_b_ > 0.2 within 1000 measurements, and *m*_b_ is the average *R*_b_ of spectra with *R*_b_ > 0.2. If not specified, *p*-values for all combinations were 〈 0.01. * means 0.05 〉 *p*-value > 0.01.

	***C*_PE_**	***R***	***R*_b_**	***H*_b_**	***H*_b_**	***H*_b_**	***H*_b_**	***H*_b_**	***H*_b_**	***H*_b_**	***f*_b_**	***m*_b_**
				@2878	@2844	@1415	@1239	@1415	@1239	@1128		
***C*_PE_**	1.00	0.73	0.85	0.63	0.59	0.75	0.76	0.57	0.69	0.67	0.81	0.26*
***R***	0.73	1.00	0.95	0.96	0.95	0.92	0.87	0.79	0.96	0.96	0.96	0.57
***R*_b_**	0.85	0.95	1.00	0.94	0.92	0.94	0.91	0.80	0.95	0.95	0.96	0.48
***H*_b_**@2878	0.63	0.96	0.94	1.00	1.00	0.91	0.86	0.81	0.98	0.99	0.91	0.58
***H*_b_**@2844	0.59	0.95	0.92	1.00	1.00	0.89	0.83	0.80	0.96	0.98	0.89	0.60
***H*_b_**@1415	0.75	0.92	0.94	0.91	0.89	1.00	0.99	0.86	0.97	0.96	0.93	0.59
***H*_b_**@1239	0.76	0.87	0.91	0.86	0.83	0.99	1.00	0.85	0.94	0.92	0.90	0.58
***H*_b_**@1415	0.57	0.79	0.80	0.81	0.80	0.86	0.85	1.00	0.85	0.86	0.79	0.56
***H*_b_**@1239	0.69	0.96	0.95	0.98	0.96	0.97	0.94	0.85	1.00	0.99	0.93	0.58
***H*_b_**@1128	0.67	0.96	0.95	0.99	0.98	0.96	0.92	0.86	0.99	1.00	0.93	0.59
***f*_b_**	0.81	0.96	0.96	0.91	0.89	0.93	0.90	0.79	0.93	0.93	1.00	0.54
***m*_b_**	0.26*	0.57	0.48	0.58	0.60	0.59	0.58	0.56	0.58	0.59	0.54	1.00

### Challenges and future directions

In this study, we prepared artificial SMP suspension samples by dissolving standard SMP samples in DI water to illustrate and validate our proposed method. However, this validation experiment still deviates from the practical application of environmental SMP samples in sea waters. In this section, we will highlight the challenges this method faces and potential strategies for overcoming these issues.

A primary challenge is that environmental SMP particles often carry surface attachments and are subjected to weathering or degradation, which can reduce spectral quality and modify spectral shape [38]. This influence may complicate the accurate identification of polymer types. Additionally, sea waters frequently contain other constituents, such as dissolved organic matter [39], which could potentially interfere with SMP polymer type identification. To counter these issues, it may be beneficial to filter sea water to remove all suspensions before proceeding with a measurement. The obtained average spectral data from the filtered water sample could then serve as a background and be utilized to correct the original sample spectra. Further exploration into optimal Raman parameters for measurement is also necessary, aiming to secure high-quality spectra while ensuring that measurements can be conducted within a reasonable timeframe.

Another challenge is that our study utilized SMPs of a single polymer type. However, in actual environmental sample analyses, multiple polymer types coexist [40]. While the average spectra of other polymer types are also anticipated to correlate with concentration, the specific interactions between multiple types remain unknown. Therefore, further research is needed to understand the behavior of average spectra in the presence of different polymer types. Moreover, the development of algorithms capable of decomposing the mixed spectra of multiple polymer types is also important.

Additionally, SMPs composed of diverse polymer types may exhibit different densities, and the actual densities become even more complex when considering environmental samples subjected to different extents of degradation and attached impurities [41]. Consequently, the relative density of SMPs to water may have influence whether the SMP particles can remain at the water surface. Therefore, further investigation of the impact of relative density on SMP distribution within water bodies is necessary. If necessary, some pretreatments, such as density adjustment, may be implemented to optimize such process.

Finally, while our study was aimed at analyzing seawater, this method may also hold potential for application in other water bodies, such as freshwater or wastewater. However, these environments present unique challenges due to their distinct compositions and potential pollutant profiles, which may significantly impact the average spectrum. Moreover, the variation in organic and inorganic constituents across these water types may affect the accuracy and reliability of polymer identification. Therefore, comprehensive studies exploring these differing water matrices and their impacts on SMP detection and characterization are necessary, along with adaptations of the method to cater to the specific needs of each water type.

## Related research article

None.

## CRediT authorship contribution statement

**Zijiang Yang:** Conceptualization, Methodology, Software, Validation, Formal analysis, Investigation, Writing – original draft, Visualization. **Hisayuki Arakawa:** Conceptualization, Resources, Writing – review & editing, Supervision, Project administration, Funding acquisition.

## Declaration of Competing Interest

The authors declare that they have no known competing financial interests or personal relationships that could have appeared to influence the work reported in this paper.
